# Evaluation of hepatectomy and palliative local treatments for gastric cancer patients with liver metastases: a propensity score matching analysis

**DOI:** 10.18632/oncotarget.18709

**Published:** 2017-06-27

**Authors:** Jiyang Li, Kecheng Zhang, Yunhe Gao, Hongqing Xi, Jianxin Cui, Wenquan Liang, Aizhen Cai, Bo Wei, Lin Chen

**Affiliations:** ^1^ Department of General Surgery, Chinese People’s Liberation Army General Hospital, Beijing 100853, China

**Keywords:** stomach neoplasms, liver, hepatectomy, radiofrequency ablation, transarterial chemoembolization

## Abstract

**Background:**

The optimal treatments for gastric cancer with liver metastases (GCLM) remain controversial. This study aimed to evaluate the efficacy of hepatectomy, RFA and TACE as local treatments for GCLM.

**Methods:**

From 2001 to 2015, 119 consecutive patients who received multidisciplinary treatments based on curative gastrectomy and local treatments (hepatectomy, RFA and TACE) for liver metastases were enrolled in this retrospective cohort study. Patients were divided into Group A (46, hepatectomy) and Group B (73, either or both RFA and TACE). Propensity score matching analysis was employed.

**Results:**

The propensity model revealed that hepatectomy was associated with significantly longer OS compared with either or both RFA and TACE (P=0.021). The 1-, 3- and 5-year OS rates were 80.5%, 41.5% and 24.4%, respectively in Group A; and 85.4%, 21.9% and 12.2%, respectively in Group B. Subgroup analyses indicated that hepatectomy was associated with significantly longer long-term survival compared with TACE (P=0.033) and RFA (P=0.010). TACE had a similar efficacy as RFA (P=0.518), but with significantly lower costs (P=0.014) in for patients with metachronous GCLM.

**Conclusion:**

Hepatectomy is the optimal local treatment for GCLM when surgical R0 resection is intended. TACE attained a similar prognosis as RFA with relatively high cost-effectiveness, particularly for patients with metachronous GCLM.

## INTRODUCTION

Gastric cancer is the fourth most common tumor and the second most common cause of cancer-related death worldwide; the highest incidence is in Eastern Asia, where approximately 1,000,000 people per year are affected [1, 2]. Hematogenous dissemination is one of the main methods by which gastric cancer metastasizes; the liver is the organ most frequently involved, with an incidence of 30%-50% [3]. Moreover, at the time of diagnosis, approximately 35% of patients have distant metastases, while 4%-14% have metastatic disease in the liver [4]. Gastric cancer liver metastases (GCLM) are associated with shorter survival [5, 6]. Surgical techniques and perioperative management have improved, and many patients with GCLM benefit from surgery [7–9]. The Japanese working group reached the conclusion that hepatectomy should be considered for carefully selected patients with GCLM[10]. To some extent, complete surgical resection is the only form of therapy with curative intent for GCLM.

Unlike colorectal liver metastasis and because of the aggressively infiltrative biological behavior of gastric cancer, liver metastases from gastric cancer are mostly characterized by multiple lesions that are diffusely distributed on both hepatic lobes and are combined with peritoneal dissemination, lymph nodes, or other distant organ metastases [6, 11]. Only 0.3%-2.4% of patients with GCLM are candidates for hepatic resection [4]. Therefore, the optimal local treatment for liver metastases remains controversial. Radiofrequency ablation (RFA) and transarterial chemoembolization (TACE) are effective and low risk, with more expanded indications in patients with liver metastases. Further, RFA and TACE can repeatedly administered to gastric cancer patients with unresectable liver metastases [12, 13].

Although a randomized controlled trial is best to evaluate the curative effect of therapy, it is unlikely to recruit patients who would agree to random assignment to complicated procedures with obvious differences. Propensity score matching (PSM) analysis can overcome selection bias, to the extent possible, to increase the evidence level of a nonrandomized observational study [14].

It is therefore imperative to use the PSM model to evaluate different methods for the local treatment of liver metastases in patients with gastric cancer. In the present study, we assessed the optimal treatment for GCLM by conducting an analysis of a cohort of consecutive patients who underwent gastrectomy accompanied by local treatment for liver metastases.

## RESULTS

### Baseline characteristics

The 119 patients enrolled in the study were all Han Chinese, 101 men and 18 women (5.6:1) with a mean age of 58.4 years (range, 20–83 years). Their occupations included public service (28, 23.5%), professional technicians (11, 9.2%), commercial staff (19, 16.0%), production staff (27, 22.7%), military (9, 7.6%) and others (25, 21.0%). Their educational levels were as follows: illiterate (0, 0%), elementary education or above (12, 10.1%), secondary education or above (79, 66.4%), and bachelor’s degree or above (28, 23.5%). Marital status was as follows: married (95, 79.8%), loss of spouse (16, 13.4%), divorced (7, 5.9%), and unmarried (1, 0.9%).

Data for the baseline clinicopathologic variables available in the database were as follows: sex, age, body mass index, Karnofsky Performance Scale (KPS) score, characteristics of the primary gastric tumor (location, size, Borrmann classification, degree of histologic differentiation, depth of invasion, lymph node metastasis), characteristics of hepatic metastases (type, number, size and distribution), neutrophil to lymphocyte ratio (NLR), presence of carcinoembryonic antigen (CEA), alpha-fetoprotein (AFP) level, and types of treatment. Baseline characteristics of all patients before matching are summarized in Table 1 . Patients with GCLM who underwent surgical resection (Group A) were significantly younger and had higher AFP levels compared with those of the patients administered palliative local treatments, RFA, TACE, or both (Group B) (Both P<0.05). Group B included more patients with metachronous liver metastases compared with those in Group A (32 vs 6, P<0.001). PSM identified 41 patients from each treatment group with similar characteristics (all P>0.05; Table 2 ).

**Table 1:** Baseline characteristics of the whole patient cohort

Characteristics	Group A (n=46)	Group B (n=73)	P-value
Age(years)*	54.9±1.6	60.0±1.3	**0.014**
Sex			0.110
Male	36(78.3%)	65(89.0%)	
Female	10(21.7%)	8(11.0%)	
BMI(kg/m2)*	23.3±0.6	23.3±0.5	0.993
KPS scores			0.222
80-	1(2.2%)	5(6.9%)	
90-	45(97.8%)	68(93.1%)	
Gastric primary tumor location			0.488
Proximal	12(26.1%)	18(24.7%)	
Middle	12(26.1%)	16(21.9%)	
Distal	17(37.0%)	23(31.5%)	
Total	5(10.8%)	16(21.9%)	
Size of gastric primary tumor(cm)^#^	5.7±0.6	5.2±0.4	0.447
Bormmann			0.641
Mass	2(4.3%)	7(9.6%)	
Ulcerative	29(63.0%)	48(65.8%)	
Infiltrative ulcerative	12(26.2%)	16(21.9%)	
Diffuse Infiltrative	3(6.5%)	2(2.7%)	
Degree of histologic differentiation			0.234
Well or moderately	26(56.5%)	28(38.4%)	
Poorly or signet-ring cell	20(43.5%)	45(61.6%)	
T			0.976
T1	3(6.5%)	7(9.7%)	
T2	3(6.5%)	5(6.8%)	
T3	3(6.5%)	5(6.8%)	
T4	37(80.5%)	56(76.7%)	
N			0.219
N0	5(10.9%)	16(21.9%)	
N1	8(17.4%)	19(26.0%)	
N2	12(26.1%)	14(19.2%)	
N3	21(45.6%)	24(32.9%)	
Type of liver metastases			
Synchronous	40(87.0%)	41(56.2%)	**<0.001**
Metachronous	6(13.0%)	32(43.8%)	
Number of liver metastases			0.394
Isolated metastases	18(39.1%)	23(31.5%)	
Multiple metastases	28(60.9%)	50(68.5%)	
H			0.140
H1	23(50.0%)	28(38.4%)	
H2	6(13.0%)	5(6.8%)	
H3	17(37.0%)	40(54.8%)	
Size of liver metastases (cm)^#^	3.8±0.5	3.2±0.3	0.255
NLR^#^	3.5±0.9	4.2±0.8	0.620
CEA (ug/L)^#^	18.4±5.6	11.2±4.7	0.329
AFP (ug/L)^#^	41.8±20.1	4.0±0.6	**0.031**
Chemotherapy			0.537
Postoperative chemotherapy	35(76.1%)	59(80.8%)	
Perioperative chemotherapy	11(23.9%)	14(19.2%)	

**Table 2:** Baseline characteristics of patients in the matched cohort

Characteristics	Group A (n=41)	Group B (n=41)	P-value
Age(years)*	54.2±1.7	58.1±1.6	0.110
Sex			0.194
Male	33(80.5%)	38(92.7%)	
Female	8(19.5%)	3(7.3%)	
BMI(kg/m2)*	22.9±0.6	24.3±0.6	0.118
KPS scores			1.000
80-	1(2.4%)	0(0)	
90-	40(97.6%)	41(100%)	
Gastric primary tumor location			0.886
Proximal	10(24.4%)	11(26.8%)	
Middle	11(26.8%)	8(19.5%)	
Distal	15(36.6%)	16(39.1%)	
Total	5(12.2%)	6(14.6%)	
Size of gastric primary tumor(cm)^#^	5.5±0.7	5.2±0.5	0.682
Bormmann			0.671
Mass	2(4.9%)	4(9.7%)	
Ulcerative	27(65.8%)	28(68.3%)	
Infiltrative ulcerative	9(22.0%)	7(17.1%)	
Diffuse infiltrative	3(7.3%)	2(4.9%)	
Degree of histologic differentiation			0.098
Well or moderately	20(48.8%)	12(29.3%)	
Poorly or signet-ring cell	21(51.2%)	29(70.7%)	
T			0.934
T1	4(9.7%)	4(9.7%)	
T2	4(9.7%)	2(4.9%)	
T3	3(7.3%)	2(4.9%)	
T4	30(73.3%)	33(80.5%)	
N			0.221
N0	5(12.2%)	8(19.5%)	
N1	8(19.5%)	14(34.2%)	
N2	11(26.8%)	11(26.8%)	
N3	17(41.5%)	8(19.5%)	
Type of liver metastases			
Synchronous	36(87.8%)	32(78.0%)	0.379
Metachronous	5(12.2%)	9(22.0%)	
Number of liver metastases			0.492
Isolated metastases	17(41.5%)	13(31.7%)	
Multiple metastases	24(58.5%)	28(68.3%)	
H			0.247
H1	20(48.8%)	16(39.0%)	
H2	6(14.6%)	3(7.3%)	
H3	15(36.6%)	22(53.7%)	
Size of liver metastases (cm)^#^	3.6±0.6	3.1±0.4	0.426
NLR^#^	2.6±0.3	3.7±0.7	0.155
CEA (ug/L)^#^	19.9±6.9	13.7±6.4	0.519
AFP (ug/L)^#^	15.1±6.8	4.7±0.8	0.084
Chemotherapy			0.806
Postoperative chemotherapy	30(73.2%)	29(70.7%)	
Perioperative chemotherapy	11(26.8%)	12(29.3%)	

Values are expressed as the mean ± SD, or number (percentage). All of the tumor-related clinicopathologic data were obtained from postoperative pathological reports. BMI, body mass index; KPS, Karnofsky Performance Scale score. Size of gastric primary tumor indicates the maximal diameter of the primary gastric tumor. Hepatic metastases were classified as H1 (limited to one lobe), H2 (a few lesions scattered in both lobes) and H3 (multiple diffusely distributed metastatic lesions in both lobes). Continuous variables: * normal distribution, Pearson chi-square test; ^#^ non-normally distribution, the Mann-Whitney test. Value shown in bold is statistically significant (P<0.05).

### Factors associated with overall survival (OS)

Univariate analysis of overall survival of all patients revealed that diffuse infiltration according to the Borrmann classification and synchronous liver metastases were significantly associated with increased mortality rates (both P<0.05; Table 3 ). The data acquired using multivariate Cox proportional hazards model revealed that only diffuse infiltration of the Borrmann classification (P=0.002; Table 3) was an independent prognostic predictor of a poor long-term outcome.

**Table 3:** Univariate and multivariate analysis for overall survival in patients.

	Univariate		Multivariate	
	HR (95%CI)	P-value	HR (95%CI)	P-value
**All patients in the whole cohort (n=119)**				
Age (year)	1.022(1.000-1.045)	0.054		
Sex		0.676		
Male	1.00 (Reference)			
Female	0.854(0.409-1.786)			
BMI(kg/m2)	1.021(0.948-1.100)	0.579		
KPS	0.996(0.915-1.083)	0.916		
Gastric primary tumor location		0.090		
Proximal	1.00 (Reference)			
Middle	0.539(0.275-1.056)	0.072		
Distal	0.575(0.319-1.036)	0.065		
Total	1.086(0.537-2.199)	0.818		
Size of gastric primary tumor (cm)	0.985(0.912-1.064)	0.698		
Bormmann		**<0.001**		**<0.001**
Mass	1.00 (Reference)		1.00 (Reference)	
Ulcerative	0.971(0.298-3.165)	0.961	0.971(0.298-3.166)	0.961
Infiltrative ulcerative	2.056(0.587-7.194)	0.260	2.058(0.587-7.207)	0.259
Diffuse infiltrative	11.281(2.363-53.860)	**0.002**	11.321(2.352-54.492)	**0.002**
Degree of histologic differentiation		0.165		
Well or moderately	1.00 (Reference)			
Poorly or signet-ring cell	1.424(0.865-2.345)			
T		0.828		
T1	1.00 (Reference)			
T2	1.244(0.350-4.413)	0.736		
T3	1.737(0.486-6.212)	0.396		
T4	1.409(0.604-3.284)	0.427		
N		0.554		
N0	1.00 (Reference)			
N1	0.895(0.425-1.882)	0.770		
N2	1.407(0.692-2.861)	0.345		
N3	1.249(0.623-2.503)	0.531		
Type of liver metastases		**0.045**		0.964
Synchronous	1.00 (Reference)		1.00 (Reference)	
Metachronous	0.567(0.325-0.991)		1.013(0.564-1.821)	
Interval of metachronous	0.970(0.940-1.002)	0.062		
Number of liver metastases		0.757		
Isolated metastases	1.00 (Reference)			
Multiple metastases	0.924(0.562-1.521)			
H		0.474		
H1	1.00 (Reference)			
H2	1.483(0.714-3.028)	0.291		
H3	0.958(0.578-1.589)	0.868		
Size of liver metastases (cm)	0.973(0.866-1.093)	0.645		
NLR	0.951(0.798-1.134)	0.579		
CEA	0.995(0.977-1.013)	0.604		
RFA	1.004(0.996-1.013)	0.330		
Chemotherapy		0.382		
Perioperative chemotherapy	1.00 (Reference)			
Postoperative chemotherapy	1.278(0.737-2.218)			
Hepatectomy	0.635(0.390-1.032)	0.067		
RFA	1.033(0.616-1.733)	0.903		
TACE	1.395(0.857-2.270)	0.180		
**Patients in the matched cohort (n=82)**				
Age (year)	1.020(0.995-1.046)	0.113		
Sex		0.626		
Male	1.00 (Reference)			
Female	0.810(0.347-1.890)			
BMI(kg/m2)	1.013(0.932-1.102)	0.756		
KPS	1.018(0.907-1.143)	0.761		
Gastric primary tumor location		**0.045**		0.211
Proximal	1.00 (Reference)		1.00 (Reference)	
Middle	0.772(0.374-1.596)	0.485	1.052(0.472-2.344)	0.901
Distal	0.579(0.301-1.112)	0.101	0.582(0.291-1.161)	0.125
Total	1.705(0.777-3.741)	0.183	1.356(0.465-3.952)	0.577
Size of gastric primary tumor (cm)	0.989(0.914-1.069)	0.778		
Bormmann		**<0.001**		**0.005**
Mass	1.00 (Reference)		1.00 (Reference)	
Ulcerative	0.860(0.261-2.830)	0.803	0.957(0.279-3.285)	0.944
Infiltrative ulcerative	2.003(0.558-7.189)	0.287	2.422(0.622-9.421)	0.202
Diffuse infiltrative	10.967(2.241-53.665)	**0.003**	8.295(1.324-51.981)	**0.024**
Degree of histologic differentiation		0.365		
Well or moderately	1.00 (Reference)			
Poorly or signet-ring cell	1.288(0.744-2.229)			
T		0.703		
T1	1.00 (Reference)			
T2	1.150(0.257-5.148)	0.855		
T3	1.966(0.437-8.857)	0.379		
T4	1.669(0.600-4.642)	0.326		
N		0.473		
N0	1.00 (Reference)			
N1	0.942(0.402-2.206)	0.891		
N2	1.551(0.693-3.469)	0.285		
N3	1.400(0.622-3.152)	0.417		
Type of liver metastases		0.445		
Synchronous	1.00 (Reference)			
Metachronous	0.767(0.388-1.516)			
Interval of metachronous	0.975(0.946-1.005)	0.096		
Number of liver metastases		0.937		
Isolated metastases	1.00 (Reference)			
Multiple metastases	0.979(0.579-1.656)			
H		0.530		
H1	1.00 (Reference)			
H2	1.583(0.709-3.534)	0.262		
H3	1.086(0.630-1.873)	0.766		
Size of liver metastases (cm)	1.000(0.889-1.125)	0.999		
NLR	1.025(0.818-1.285)	0.828		
CEA	0.992(0.973-1.011)	0.416		
AFP	1.016(0.985-1.048)	0.313		
Chemotherapy		0.514		
Perioperative chemotherapy	1.00 (Reference)			
Postoperative chemotherapy	1.209(0.684-2.135)			
Hepatectomy	0.553(0.329-0.929)	**0.025**	0.560(0.312-1.004)	0.052
RFA	1.069(0.603-1.069)	0.820		
TACE	1.558(0.923-2.631)	0.097		

HR, hazard ratio; CI, confidence interval; NLR, neutrophil-to-lymphocyte ratio; CEA, carcinoembryonic antigen; AFP, alpha-fetoprotein

Apart from diffuse infiltration of the Borrmann classification (P=0.003) as an adverse effect, univariate analysis of the matched cohort revealed that hepatectomy was associated with shorter survival. Further, the location of the primary gastric tumor significantly influenced prognosis (P=0.045; Table 3), although this was excluded by the results of analysis using the Cox proportional hazards model (P>0.05; Table 3). The protective effect of hepatectomy was uncertain with a marginal value P=0.052 (Table 3). Diffuse infiltration of the Borrmann classification (P=0.024; Table 3) was an independent adverse prognostic predictor of OS.

### Survival analysis

#### All patients

**Figure 1:**
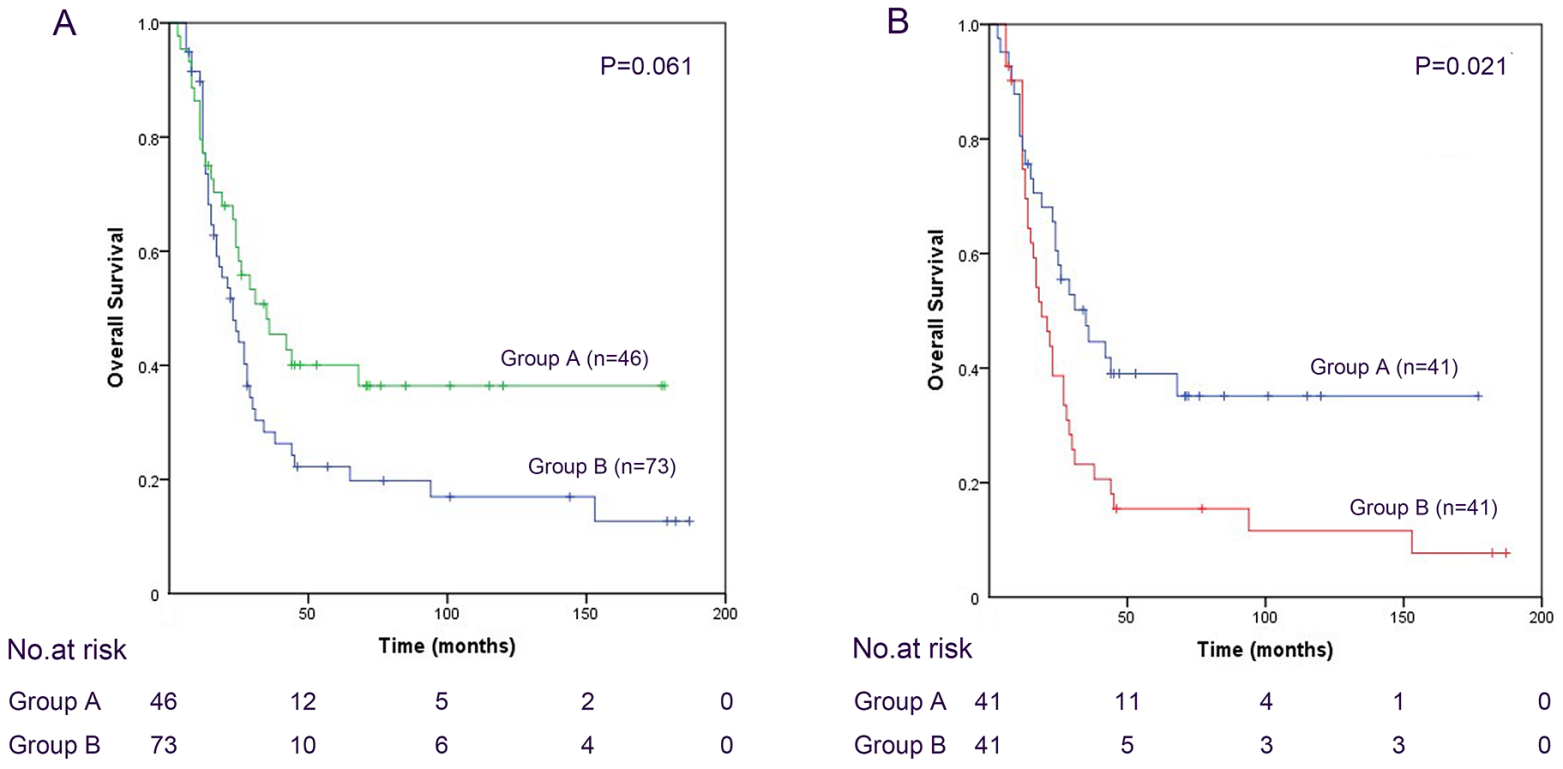
Cumulative overall survival was analyzed using the Kaplan-Meier method, and the differences in survival curves among the groups were compared using the log-rank test **(A)** Overall survival of the 119 patients in Group A and Group B before propensity score matching analysis. Kaplan-Meier survival analysis revealed similar overall survival between Groups A and B. (χ2=3.514; P=0.061). **(B)** Overall survival of the 82 patients in Group A and Group B after propensity score matching analysis. Using PSM revealed that patients who underwent hepatectomy had significantly longer overall survival compared with those received palliative local treatments. (χ2=5.289; P=0.021)

The mean overall survival time (OST) of the 119 patients was 40 months (range, 3–187 months), 43 months (range, 3–178 months) for the 46 patients in Group A and 37 months (range, 6–187 months) for the 73 patients in Group B. The 1-, 3- and 5-year survival rates were 79.5%, 40.9%, and 25.0%, respectively, in Group A and 84.7%, 23.7% and 15.3%, respectively, in Group B. Kaplan-Meier survival analysis revealed similar OST between Groups A and B (χ^2^=3.514; P=0.061; Figure 1A). There was no treatment-related mortality.

#### Matched cohort

Using PSM revealed that patients who underwent hepatectomy had significantly longer OST compared with those received palliative local treatments (χ^2^=5.289; P=0.021; Figure 1B). The 1-, 3- and 5-year OS rates were 80.5%, 41.5%, and 24.4%, respectively, in Group A and 85.4%, 21.9% and 12.2%, respectively, in Group B.

#### Subgroup analysis

**Figure 2:**
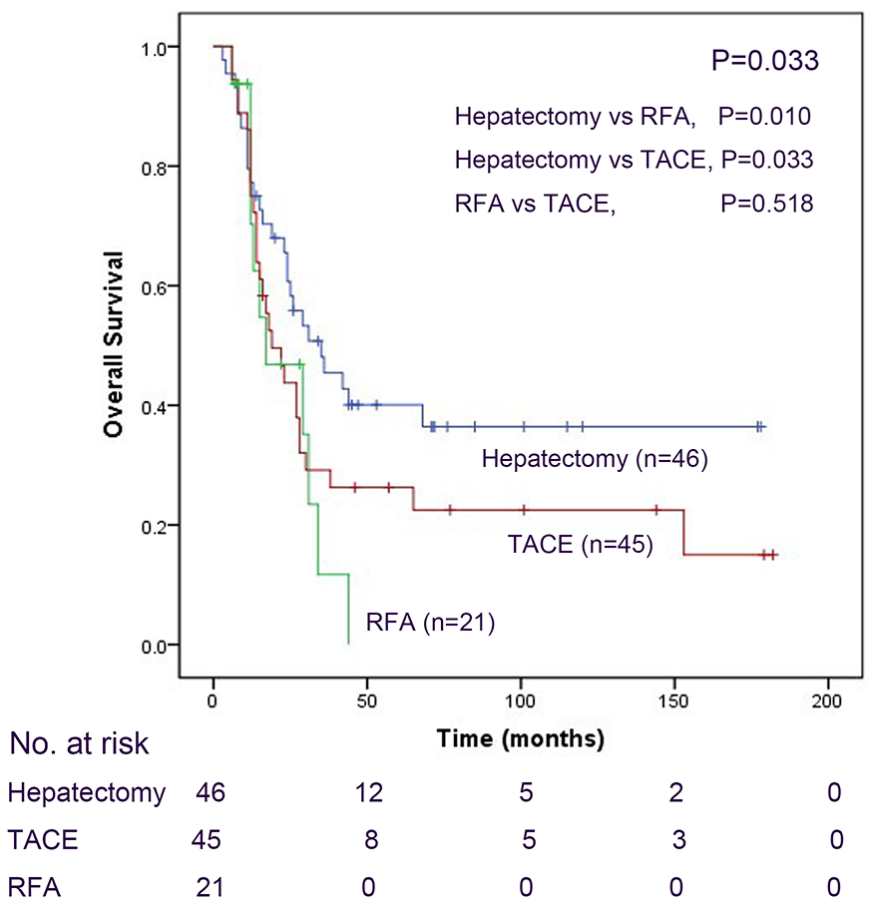
We compared the long-term outcomes of patients who received a single local treatment (hepatectomy, 46 patients; TACE, 45 patients; and RFA, 21 patients) using the Kaplan-Meier method and the log-rank test to evaluate the differences between groups

**Figure 3:**
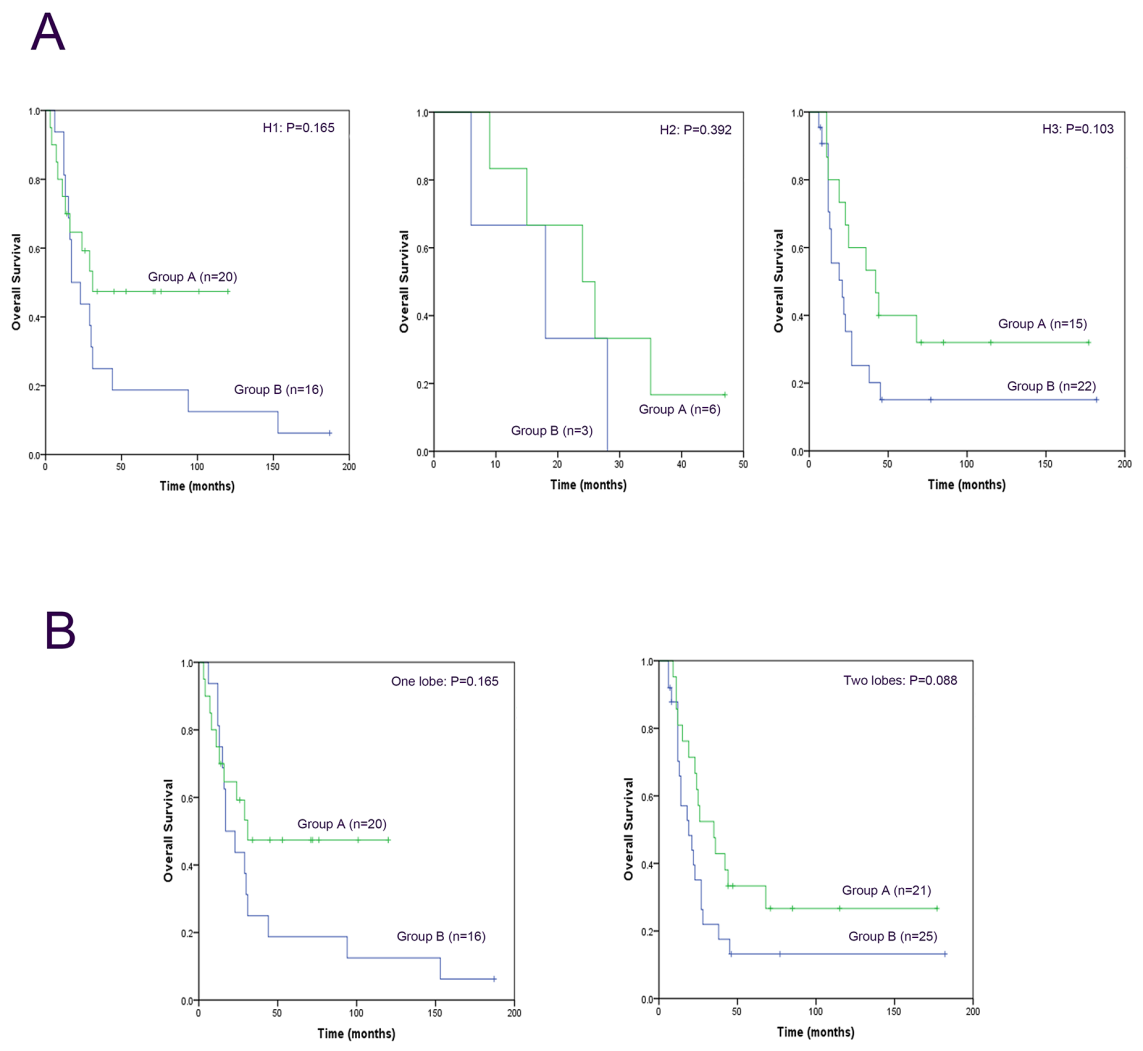
Cumulative overall survival was analyzed using the Kaplan-Meier method, and the differences in survival curves among the groups were compared using the log-rank test **(A)** The long-term outcomes of patients in Group A and Group B were separately compared in the H1, H2 and H3 subgroups. **(B)** The long-term outcomes of patients in Group A and Group B were separately compared with respect to different degrees of liver metastases (one lobe vs two lobe) subgroups.

To better explain the effect of these three local treatments, we compared the long-term outcomes of patients who received a single local treatment (hepatectomy, 46 patients; TACE, 45 patients; and RFA, 21 patients). The mean OST was 70 months for the 46 patients who underwent hepatectomy, 54 months for the 45 patients received TACE, and 23 months for the 21 patients received RFA. The actuarial 1-, 3- and 5-year survival rates were 84.0%, 52.0% and 28.0%, respectively, for hepatectomy; 86.1%, 27.8% and 19.4%, respectively, for TACE; and 75%, 6,3% and 0, respectively, for RFA. Hepatectomy achieved significantly longer survival compared with the other two palliative local treatments (χ^2^=6.843; P=0.033; Figure 2). Comparison of the groups led to the same conclusion as follows: hepatectomy was significantly associated with OS compared with TACE (χ^2^=4.538; P=0.033; Figure 2) and RFA (χ^2^=6.647; P=0.010; Figure 2). The efficacy of TACE was similar to that of RFA (χ^2^=0.418; P=0.518; Figure 2). The distribution of liver metastases is an important factor that influences prognosis. Therefore, we performed separate comparisons of the long-term outcomes of patients in Groups A and B in the H1, H2 and H3 subgroups. There were no statistical differences in the long-term survival of the subgroups (H1, χ^2^=1.926, P=0.165; H2, χ^2^=0.732, P=0.392; H3, χ^2^=2.663, P=0.103; Figure 3A), which may be explained by the small number of patients in each subgroup (H1=36, H2=9, H3=37). Further, we found that the degree of liver metastases (one lobe vs two lobes) did not affect the prognosis of patients with GCLM in Groups A and B (one lobe, χ^2^=1.926, P=0.165; two lobes, χ^2^=2.919, P=0.088; Figure 3B).

### Financial cost

We calculated the average treatment costs for patients with synchronous GCLM who received gastrectomy combined with hepatectomy, RFA, or both and patients with metachronous GCLM who received TACE, RFA or both as local treatments for liver metastases (Table 4). The results indicate that financial costs were similar for patients with synchronous GCLM (P=0.164). However, TACE was significantly more cost-effective for patients with metachronous GCLM (TACE = 39,215 RMB and RFA = 95,250 RMB, P=0.014).

**Table 4:** Financial costs of treatment

	Average cost (RMB, yuan)	P-value
Synchronous		0.164
Gastrectomy+hepatectomy	81488±17161	
Gastrectomy+RFA	118584±60365	
Gastrectomy+hepatectomy+RFA	113383±21962	
Metachronous		**0.014**
TACE	39215±12598	
RFA	95250±42129	
TACE+RFA	88265±38287	

Bold value is statistically significant (P<0.05). TACE, transarterial chemoembolization; RFA, radiofrequency ablation

## DISCUSSION

Oncologists have become increasingly interested in local treatments of patients with GCLM. Hepatectomy, RFA and TACE are all valuable therapeutic procedures. However, very limited data are available regarding the optimal management strategy. Here we investigated a cohort of gastric cancer patients with liver metastases only to clarify the effect of local treatments selection on long-term outcomes. Our results reveal that radical hepatectomy was very effective for treating certain patients with GCLM. TACE, an alternative local treatment, attained a similar prognosis as RFA with relatively high cost-effectiveness, particularly in metachronous GCLM patients.

The initial analysis of the entire cohort did not detect a significant difference between hepatectomy and palliative local treatments (RFA, TACE or both). Noticeably, the hepatectomy group included fewer patients with metachronous liver metastases, more patients with younger age or higher AFP levels compared with that of the palliative local treatment group. Therefore, we conducted PSM analysis to minimize these potential confusion. In the propensity model, we verified that hepatectomy was the optimal therapy for patients with GCLM.

However, hepatectomy is not suitable for every patient with GCLM. Unlike colorectal liver metastases, [15, 16] only a minority of GCLM patients are candidates for hepatic resection and most of them are synchronous GCLM patients. The severe adhesions in the upper abdominal organs caused by previous surgery often leads to high mortality and morbidity of patients who undergo metachronous resection. Therefore, it is crucial to identify suitable candidates for liver resection. According to our experience, we recommend that the indications for hepatectomy include the following: (a) preoperative imaging showing no signs of local aggression, peritoneal dissemination, or extrahepatic metastasis; (b) resectable primary gastric tumor and liver metastases, leaving a negative margin; and (c) acceptable hepatic function indicated by the serum liver function test panel. If hepatic resection is impossible to achieve because of the location, size or number of liver metastases, RFA and TACE should be considered.

RFA has been reported to have been used in well selected GCLM patients with solitary lesions of <3 cm in diameter;[17, 18] particularly as a supplementary treatment for hepatectomy in cases with borderline resectability [19]. However, a study of a small sample size[20] reported that because of multiple intrahepatic recurrence, RFA is not recommended as an independent therapy for hepatic metastases from gastric cancer. RFA should therefore be considered more suitable for combination therapy.

Compared with systemic chemotherapy, TACE has the unique advantage for delivering high-concentration drugs to metastases as well as to control systemic toxicity. A previous study[21] involving a small sample size demonstrated the effectiveness of TACE for treating GCLM. In the present study, Kaplan-Meier analysis indicated that the efficacy of TACE was similar to that of RFA. However, TACE was significantly less expensive compared with RFA when administered to patients with metachronous GCLM. With more expanded indications, we believed that TACE may be more promising if included in standardized treatments for GCLM.

Unlike previous studies, [7, 22–24] we found here that only diffuse infiltration of the Borrmann classification independently predicted prognosis. Other factors, such as serosal invasion of the primary gastric cancer, number and size of hepatic metastases, and tumor-free margin did not significantly affect prognosis. This may be a reasonable result considering the development and standardization of curative resection. Moreover, multidisciplinary and comprehensive treatments have a major impact on the natural course of disease, which significantly reduces the influence of patients’ clinicopathological characteristics.

We verified findings supported by the research of Kinoshita et al [7]. and Cheon et al. [9], who found that patients with metachronous GCLM survived significantly longer compared with those with synchronous GCLM (Supplementary Figure 1). And another interesting negative conclusion got our attention. We proved that the H classification did not significantly influence OS (Supplementary Figure 2), which is in stark contrast to the findings of our preliminary study [25]. Hepatectomy should be attempted regardless of the number of liver lesions, provided that all the metastases can undergo R0 resection. We believe that this novel conclusion is inspiring, because our findings show promise for providing more therapeutic opportunities for patients with H2 or H3 liver metastases.

The scarcity of patients and the diversification of chemotherapy strategies makes it difficult to assess the effectiveness of perioperative chemotherapy. Nevertheless, preoperative chemotherapy should be carefully considered, because it theoretically helps to identify non-responders and assist the preparation of alternative strategies when surgery is futile.

Expect for the traditional local treatments (surgery, TACE and RFA), the development of radiological and imaging techniques makes radiotherapy an alternative option for patients with GCLM who have contraindications for these treatments, such as severe cirrhosis, tumors wrapped around a vulnerable structure or a complication with underlying diseases. Intensity modulated radiotherapy, the next-generation 3-dimensional conformal radiation therapy, potentially delivers higher doses to improve control and decreases toxicity by limiting dose to normal structures [26]. Volumetric modulated arc therapy, another new radiation technique, can achieve highly conformal dose distributions on target volume coverage and sparing of normal tissues [27].

The present study has several limitations. First, its retrospective nature is prone to potential bias. Even a careful designed PSM analysis cannot completely avoid bias. Second, the selection of therapy mainly depended on doctors’ and patients’ preferences. The analysis of financial costs shows that multidisciplinary synthetic therapy for GCLM is relatively expensive. With the exception of the indications for local treatment, there are other requirements, including a patient’s understanding of treatment options for late-stage cancer and economic sustainability. Another limitation is that the initial cases were from a single institution, which reduces the generalizability of the results. Therefore, more studies involving large-scale samples from multiple centers are required to further confirm the conclusions.

In summary, gastrectomy with D2 lymph node dissection is well accepted by certain patients with advanced gastric cancer, and hepatectomy is therefore the optimal local treatment for liver metastases when surgical R0 resection is intended. If patients are not appropriate for hepatectomy, palliative local treatments such as TACE and RFA are recommended. In our experience, TACE is an acceptable method with relatively high cost-effectiveness.

## METHODS

### Patients

The application of multidisciplinary synthetic therapy at the Chinese People’s Liberation Army (PLA) General Hospital, Beijing, China was initiated in January 2001. Since then, the prospective database named the Gastric Cancer with Liver Metastasis Sub-database (GCLM-SD) was developed. The GCLM-SD collects all patients with GCLM who accept multidisciplinary and comprehensive treatments strategies based on gastrectomy and local treatment options for liver metastases. Patient demographics, clinicopathological features of the primary gastric tumor and liver metastases, treatment information, perioperative parameters and survival status are recorded in the database.

By the end of 2015, 1092 gastric cancer patients with liver metastases were diagnosed at the Chinese PLA Hospital. Of all these patients, 597 patients did not received gastrectomy, 279 patients received gastrectomy but no local treatments for liver metastases. Patients were included if they met the criteria as follows: i) liver-only metastases from gastric cancer at the time of diagnosis detected abdominal ultrasonography combined with magnetic resonance imaging or computed tomography, pathological examination or surgical exploration, ii) absence of untreated second primitive malignancies before surgery, and iii) received curative gastrectomy with D2 lymph node dissection and local treatments, including hepatectomy, RFA or TACE for liver metastases. Patients were excluded if they were receiving treatment for other serious underlying diseases or malignant tumors, if they were noncompliant, or unable to complete follow-up. Accordingly, 119 patients were eligible for inclusion and were divided into Group A (46, curative hepatectomy) and Group B (73, palliative local treatments: 21, RFA; 45, TACE; 7, combined RFA and TACE).

All enrolled patients gave their informed consent. This study was approved by the institutional review board of the Chinese People’s Liberation Army General Hospital. We conducted follow-up during outpatient visits or by telephone. OS was calculated from the day of diagnosis of GCLM to the day of death or to December 2016, the last follow-up date.

### Treatments

All the included patients underwent curative gastrectomy with D2 lymph node dissection. Subtotal gastrectomy was indicated for distal gastric cancer. For proximal gastric cancer or tumors involving more than one section of the entire stomach, proximal gastrectomy or total gastrectomy was preferred. All surgeries were performed with adequate margins (≥5 cm) using the Billroth I, Billroth II or Roux-en-Y techniques for reconstruction. After surgery, resected specimens were routinely processed for pathological assessment. Hepatectomy was performed only when surgical R0 resection was intended. For patients with synchronous hepatic metastases, hepatectomy was simultaneously performed with gastrectomy. Alternatively, RFA, TACE, or both were performed as alternative therapeutic procedures to patients with unresectable liver metastases. Here the objective was to achieve maximal cytoreduction according to specific conditions. For example, for patients who had undergone a previous abdominal operation for another disease, or tumor recurrence after radical resection, it is extremely difficult to perform hepatectomy because of severe adhesions. RFA was performed in some cases where metastases were so extensive that an insufficiently functioning liver remained after R0 resection. TACE was considered when the location of liver metastases was inconvenient for surgery or RFA, such as those close to the hepatic hilum, subphrenic space, or gall-bladder. Chemotherapy was allowed before or after surgery. Three-drug or two-drug cytotoxic regimens were administered to patients with KPS scores >80 who were subject to frequent toxicity evaluations. Because of rapid advances in the development of chemotherapeutics, many combinations of these agents were proposed. However, most regimens employ 5-fluorouracil based regimens, with the next most frequently used drugs being platinum compounds, docetaxel and epirubicin. All patients selected their multidisciplinary treatments after receiving a thorough explanation of the risks and possible alternatives, including the financial costs.

### Criteria

The definition of tumor TNM-staging was based on the 3rd English Edition of the Japanese Classification of Gastric Carcinoma.[28] The size of the primary gastric tumor refers its maximal diameter. Tumor location was considered “total” if more than one section of the entire stomach was involved. Information regarding the degree of histologic differentiation was obtained from the pathological report, including well differentiated, moderately differentiated, poorly differentiated and signet-ring cell carcinoma. The NLR was calculated as follows: NLR= absolute neutrophil count/absolute lymphocyte count [29].

### Propensity score matching analysis

To investigate the association between treatment selection and long-term outcomes in an observational, non-randomized study, PSM analysis was employed to overcome possible bias in selecting patients. Possible variables associated with the selection of treatment, including age, sex, BMI, KPS scores, primary gastric tumor location, size of gastric primary tumor, Bormmann type, degree of histologic differentiation, depth of invasion (T stage), lymph node metastasis (N stage) and characteristics of liver metastases (type, number, distribution and size) were comprehensively analyzed in the baseline comparisons. Variables associated with statistically significant differences (P <0.05) were included in the PSM analysis. A one-to-one matching requirement via the nearest-neighbor matching algorithm without replacement was performed to select matched pairs of patients. The analysis was conducted by using R statistical software, version 3.2.1.

### Other statistical analyses

Continuous variables of the two groups were checked for normality of distribution using the one-sample Kolmogorov-Smirnov test, and compared using analysis of variance (Student’s t-test or the Mann-Whitney U-test). Binomial and categorical data were evaluated by using cross-linked tables and the Pearson chi-squared test or the 2-tailed Fisher’s exact test. Characteristics influencing survival were identified by means of univariate and multivariate analyses using the Cox proportional hazards model (backward stepwise entry, 0.05; removal, 0.10). Cumulative OS was analyzed using the Kaplan-Meier method, and the differences in survival curves among groups were compared using the log-rank test. Statistical significance was defined as P <0.05 (two-sided). These statistical analyses were performed using SPSS software (Version 19.0; SPSS Inc., Chicago, IL, USA).

## SUPPLEMENTARY MATERIALS FIGURES



## Abbreviations

GCLM: liver metastasis from gastric cancer
RFA: radiofrequency ablation
TACE: transarterial chemoembolization
KPS: Karnofsky Performance Scale
NLR: neutrophil to lymphocyte ratio
CEA: carcinoembryonic antigen
AFP: alpha-fetoprotein
OS: overall survival
OST: overall survival time
BMI: body mass index
HR: hazard ratio
CI: confidence interval
