# Supplementary material for: Evaluation of hepatectomy and palliative local treatments for gastric cancer patients with liver metastases: a propensity score matching analysis
Source: Oncotarget. 2017 Jun 27;8(37):61861–75. doi: 10.18632/oncotarget.18709 (PMC5617470; doi:10.18632/oncotarget.18709)
Supplement: Supplementary file 1 [file oncotarget-08-61861-s001.pdf]

Evaluation of hepatectomy and palliative local treatments for gastric cancer patients with liver metastases: a propensity score matching analysis

SUPPLEMENTARY MATERIALS

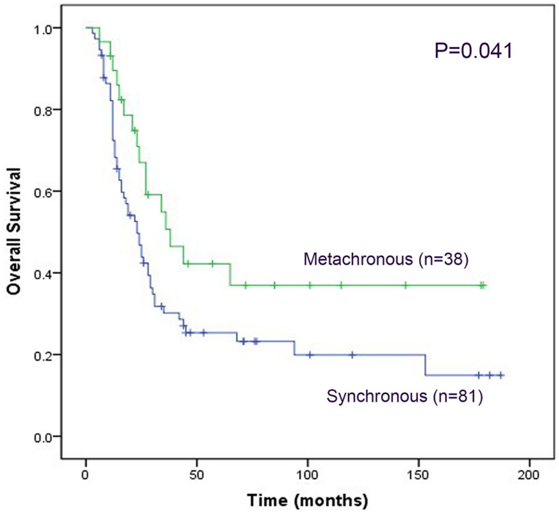

Supplementary Figure 1: Patients with metachronous GCLM survived significantly longer compared with synchronous GCLM.

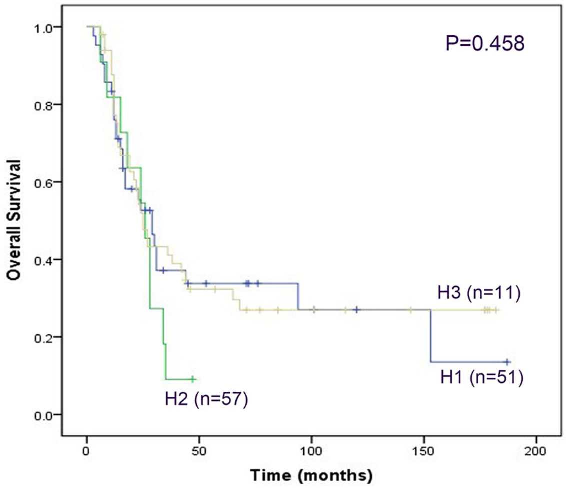

Supplementary Figure 2: The H classification did not significantly influence overall survival.
